# Oxytocin in the Male Reproductive Tract; The Therapeutic Potential of Oxytocin-Agonists and-Antagonists

**DOI:** 10.3389/fendo.2020.565731

**Published:** 2020-10-22

**Authors:** Beatrix Stadler, Michael R. Whittaker, Betty Exintaris, Ralf Middendorff

**Affiliations:** ^1^Institute of Anatomy and Cell Biology, Justus-Liebig-University, Giessen, Germany; ^2^Drug Discovery Disposition and Dynamics, Monash Institute of Pharmaceutical Sciences, Melbourne, VIC, Australia; ^3^Drug Discovery Biology, Monash Institute of Pharmaceutical Sciences, Melbourne, VIC, Australia

**Keywords:** oxytocin, male reproduction, oxytocin receptor signaling, oxytocin and arginine vasopressin crosstalk, oxytocin-agonist and antagonist, testis, epididymis, prostate

## Abstract

In this review, the role of oxytocin and oxytocin-like agents (acting via the oxytocin receptor and belonging to the oxytocin-family) in the male reproductive tract is considered. Previous research (dating back over 60 years) is revised and connected with recently found aspects of the role oxytocin plays in male reproductive health. The local expression of oxytocin and its receptor in the male reproductive tract of different species is summarized. Colocalization and possible crosstalk to other agents and receptors and their resulting effects are discussed. The role of the newly reported oxytocin focused signaling pathways in the male reproductive tract, other than mediating contractility, is critically examined. The structure and effect of the most promising oxytocin-agonists and -antagonists are reviewed for their potential in treating male disorders with origins in the male reproductive tract such as prostate diseases and ejaculatory disorders.

## Introduction

Oxytocin (OT) (Pitocin and Syntocinon) has been FDA (Food and Drug Administration) approved and used for nearly 40 years in supporting uterus contractions. The initial research into the uterotonic effect of OT in the female has been extended to research into OT's contractile potential throughout both the female and male reproductive tracts. It is only in recent years that the focus has shifted to further OT-effects such as (malignant) proliferation (1, 2), metabolism (3), central nervous system, and social behavior changes (4) resulting from additional OT-signaling pathways. Receptor binding, and its colocalization with other receptors, also appear to be important in determining oxytocin's role in different processes. Since OT shows a high homology to other non-apeptides (such as arginine vasopressin) and other OT-like agents (e.g., mesotocin) it is important to consider the role of signaling crosstalk in the target tissue (5). Although oxytocin is present in most mammals, there are species-dependent differences in relation to receptor selectivity and tissue sensitivity (6–8). Taken together, these facts may explain why, despite hundreds of compounds being synthesized, so few oxytocin-agonists and -antagonists have reached clinical testing and even fewer have been approved for clinical application (e.g., atosiban, carbetocin, and demoxytocin to date).

Since additional oxytocin signaling pathways have been recently identified and are currently being investigated, we wanted to revisit the role of OT in the male reproductive tract [last reviewed 2006 and 2007 (9, 10)]. We have integrated new findings and highlighted the potential of OT-agonists and -antagonists in addressing disorders of the male reproductive tract such as benign prostatic hyperplasia (BPH), premature ejaculation and anorgasmia.

## Male Reproductive Tract

**Figure 1:**
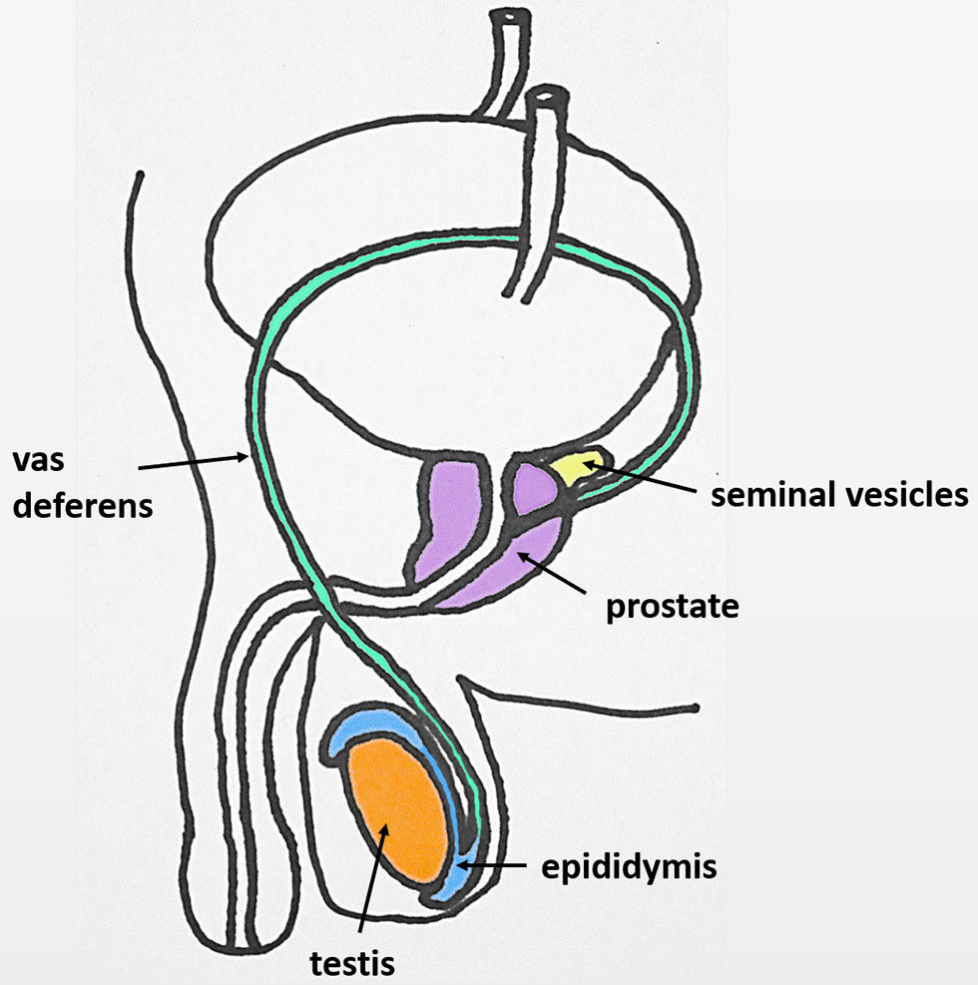
Original: Anatomy of the human male reproductive tract.

The male reproductive tract in most mammals (including the human) consists of the same organs (testis, epididymis, vas deferens, accessory sex glands, penis) (Figure 1). However, there are species-related differences with respect to anatomy, histology and the presence (or absence) of the different accessory sex glands (prostate, ampulla of vas deferens, seminal vesicles, bulbourethral glands, coagulating gland, preputial gland). Briefly, the two testes generate infertile sperm through spermatogenesis within the seminiferous tubules. These small tubules eventually unite into a single larger duct when exiting each testis, the epididymal duct, the main structure of the epididymis. The epididymis can be compartmentalized into four portions: initial segment, caput, corpus and cauda. The infertile sperm exiting the testis gain fertility and mobility as they transition through this duct to be ultimately stored as mature sperm in the very last duct segments of the cauda epididymis. The ejaculatory process can be divided into two phases: emission and expulsion. During the emission phase the sperm stored in the cauda epididymis is driven through the vas deferens into the beginning of the urethra where it is mixed with the fluids of the accessory sex glands. This seminal fluid is then expelled during the expulsion phase through the urethra out of the penis.

## Oxytocin and Its Pathway

Oxytocin (greek ȯξ$\dot{\text{u}}$ς, oxys, and τoκ*ετ*óς, toketos, meaning “quick birth”) has first been described in 1906 (by Sir Henry Dale) to have an uterotonic effect and was first synthesized in 1953 [by (11)].

**Figure 2:**
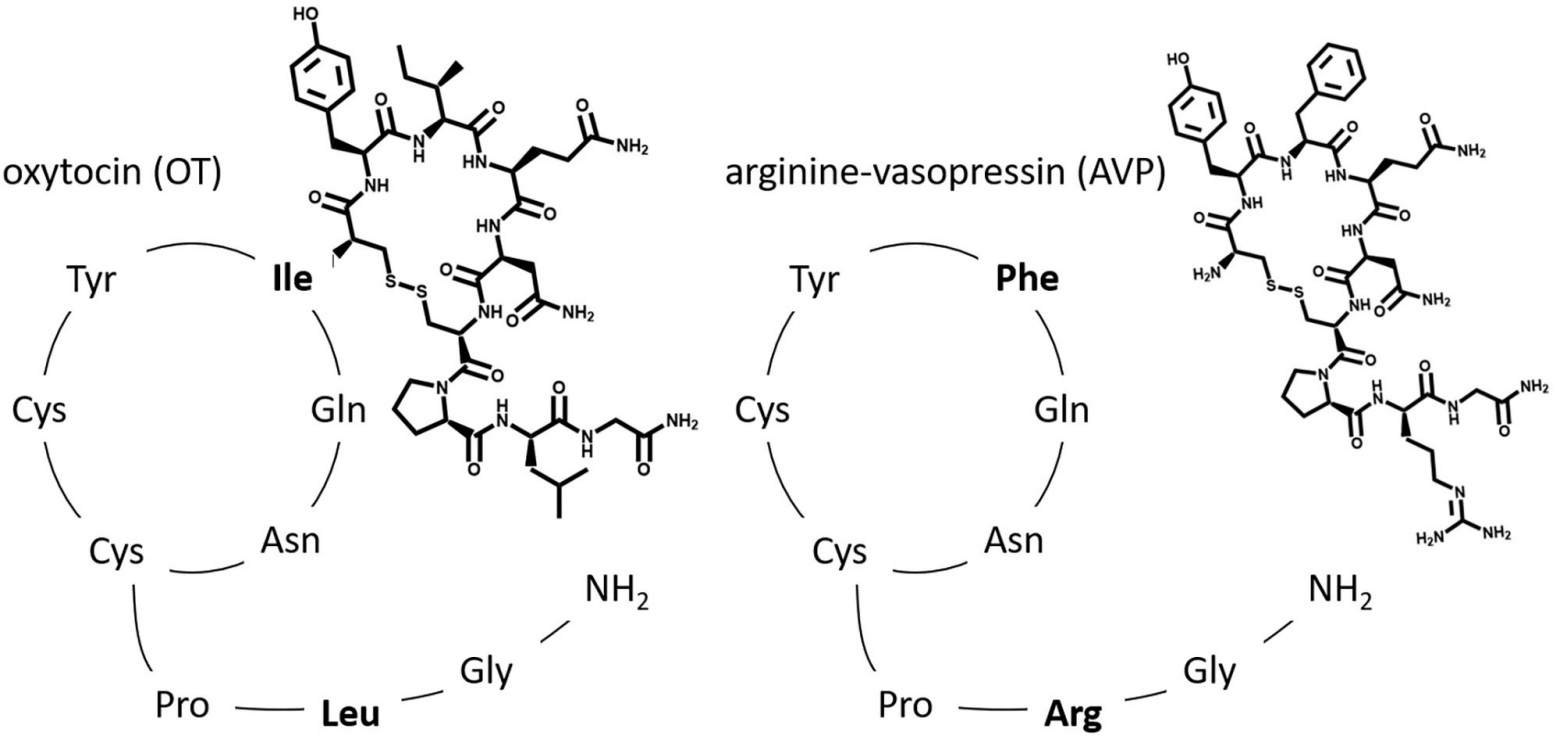
Original: The relative amino acid sequence of the two posterior pituitary nonapeptides, oxytocin (OT) and arginine vasopressin (AVP). Highlighted are the two differing amino acids found in the 3rd and 8th position.

Oxytocin (OT) and oxytocin-like peptides are nonapeptides that occur in almost all vertebrate species. The oxytocin-family has a very similar structure to the arginine vasopressin (AVP)-family (differing only in the 3rd and 8th position), allowing either nonapeptide (coming from the OT-family or the AVP-family) to crosstalk with each receptor—especially in high concentrations (5). Therefore, AVP-interactions must be considered when analyzing OT-effects (12). It is important to note that both hormones and their receptors are encoded by different genes depending on the species, and thus exhibit species dependent structural and functional differences (13). In both OT and AVP a disulfide bond between the two Cys residues at the 1st and 6th position results in a conformation of a 6 amino acid cyclic component with a 3 amino acid C-terminal part (14, 15) (Figure 2).

The oxytocin-receptor (OTR) and all three AVP-receptors (AVPR1A, AVPR1B, and AVPR2) belong to the subfamily A6 of the rhodopsin-type (class I) G protein-coupled receptors and as such they consist of seven transmembrane helices, three intra- and three extracellular loops, an extracellular N-terminus and an intracellular C-terminus (16–18).

Very recently, Waltenspühl et al. were able to report the crystal structure of the human OTR, with and without interaction of an OTR-selective antagonist (19). However, there is still much to learn about the OTR since the identification of those residues important for ligand binding have yet to be fully identified. The N-terminus of the human OTR; especially Arg^34^ (20) in the N-terminus (21); has been identified as important for agonistic binding (22–24) while not contributing to receptor selectivity between OTR and AVP-receptor. The first extracellular loop might be a major binding-site epitope by recognizing agonists and antagonists with submicromolar affinity. However, the high-affinity binding and full subtype selectivity of ligands require epitopes located in transmembrane domains. Therefore, the extracellular surface might be important for the initial “capture” of ligands prior to final “docking” to the receptor (25). Large polar residues, such as glutamine and lysine, located in transmembrane regions 2,3,4, and 6 have been proposed to be involved in the binding of the neurohypophysial hormone (26). The binding site for an OT-antagonist was found to be formed by transmembrane helices 1,2, and 7 (27). One study found that the proximal portion of the C-terminus of the OTR seems to be required for coupling to G_q_, but not G_i_ (28). The AVP1A-receptor appears to have additional glycosylation sites in the second and third loop which might distinguish it from the other AVP receptors and the OTR (12, 29). One study proposed that more interest should be paid to the development of coupling-selective analogs; molecules capable of exerting selective effects within a single receptor subtype (7). In spite of these insights, there still appears to be much to discover regarding the identification of specific ligand binding sites.

**Figure 3:**
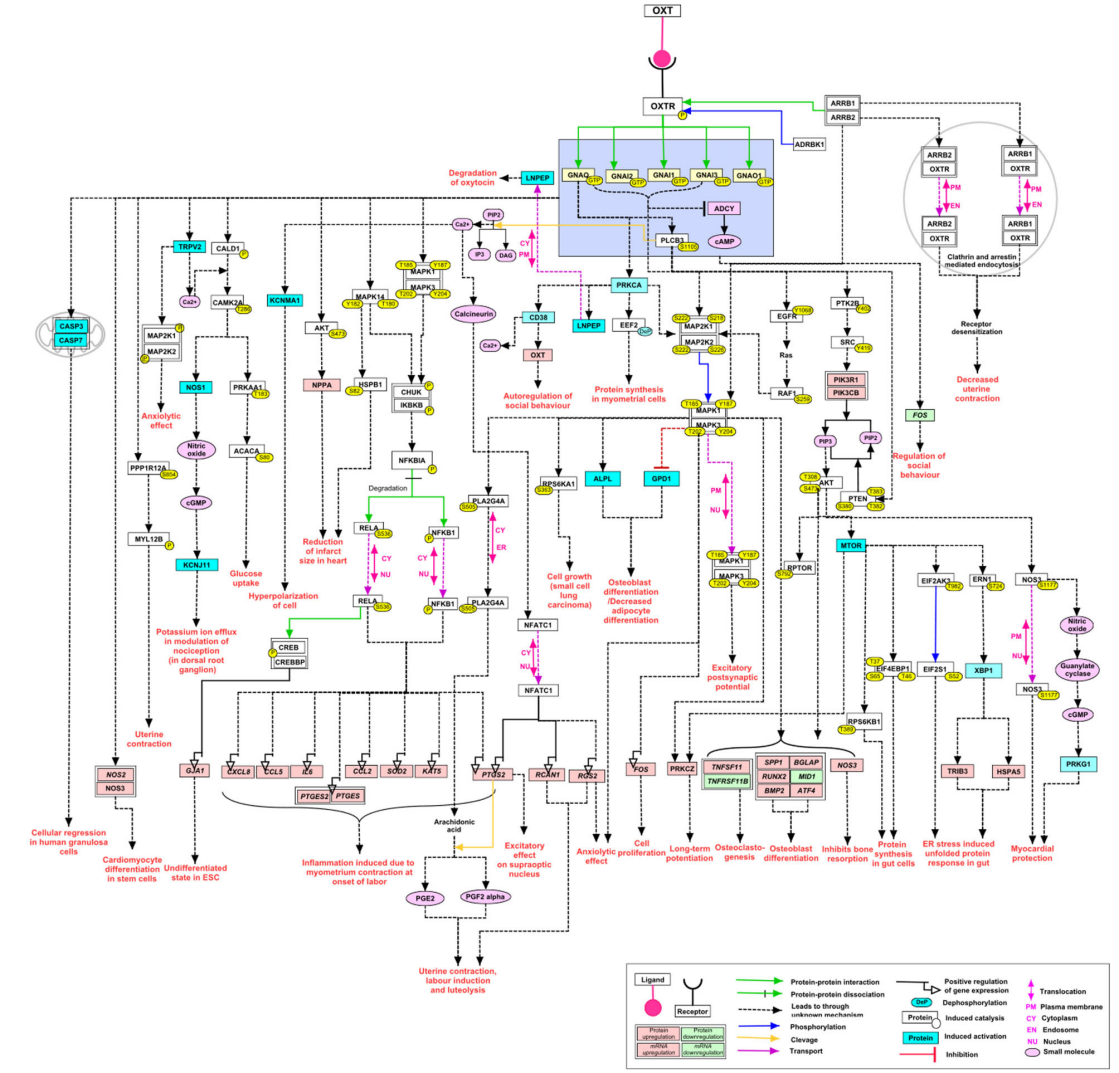
Re-published: The complexity of the signaling pathways of the oxytocin receptor (here called OXTR) Reprinted by permission from Springer Nature: Springer Link, Journal of Cell Communication and Signaling, An overview of the oxytocin-oxytocin receptor signaling network, Chatterjee et al. (33) https://link.springer.com/article/10.1007%2Fs12079-016-0353-7.

**Figure 4:**
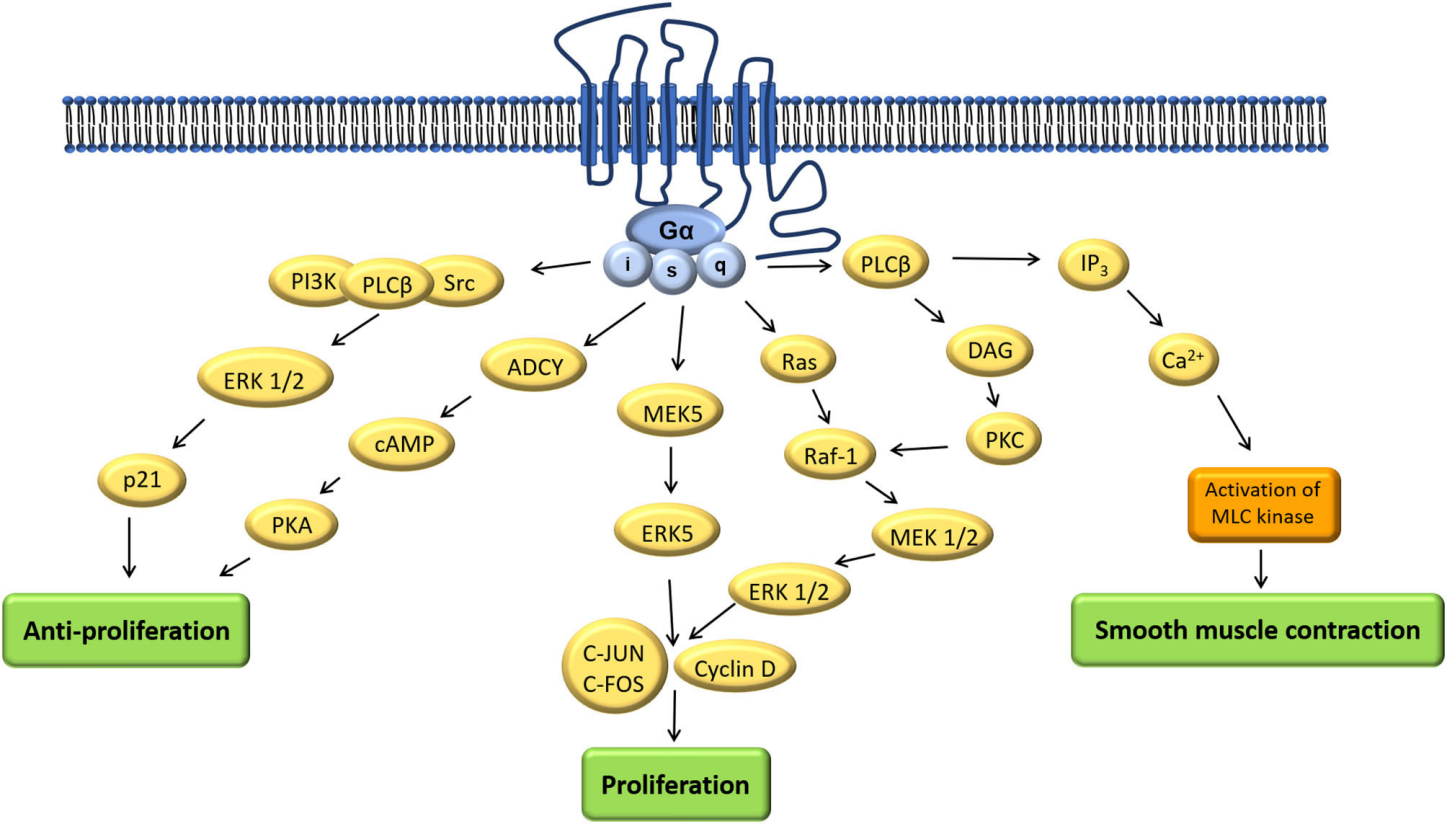
Adapted: Mechanism of actions of oxytocin via different signaling pathways (1).

Coupling to different G-protein-subunits has been found to change OTR-signaling (q, i, s) (17). Signaling pathways resulting from coupling via the Gα_q/11_-subunit (present in smooth muscle cells) have been studied the most because of their presence in the myometrium and their established contribution to myometrial contractility. Other G-protein-subunits and their signaling pathways are gaining more attention because of their supposed proliferative (30) and anti-proliferative (31, 32) effect using a multitude of signaling components (33) (Figure 3). Herein we focused on OTR signaling pathways suggested to be important in the male reproductive tract: smooth muscle contraction, and both proliferative and anti-proliferative processes (1) (Figure 4).

OTRs were found to change their downstream signaling pathway depending on their location inside or outside of caveolae. Activation of human OTR outside of caveolae resulted in inhibiting proliferation whereas inside of caveolae it resulted in promoting proliferation (34, 35). It is important to note that activation of ERK 1/2 is present in both the proliferative and the anti-proliferative pathway and the resulting effect appeared to depend on the duration of activation. This activation seemed to be more persistent when the OTRs are located outside caveolar microdomains and inhibited cell growth by activating cell cycle inhibitor p21. In contrast ERK 1/2-activity was shorter when they were located inside caveolar microdomains resulting in cell growth (34). In OT-treated cells the level of phosphorylated ERK 1/2 (compared to the total ERK 1/2) and MEK 1/2 (compared to the total MEK 1/2) in prostatic tissue was significantly higher than in the cultured cells used as a control (36). The prevalence of OTRs inside caveolae has been found to be increased in BPH-tissue (37). Both caveolin-1- and OTR-expression increase with age (37) and their colocalization increases in BPH patients (38). In human cell culture experiments OT inhibited prostatic stromal cell proliferation but had no effect on prostatic epithelial cells when cultured alone. OT by itself had no effect on malignant cells, however, in combination with testosterone it stimulated androgen independent PC-3 cell growth (2). Disruption of caveolae only removed the inhibitory effect of OT on the prostatic stromal cells but did not affect the stimulatory effect of OT on PC-3 cells cultured in the presence of androgens (2). In prostate cancer tissue and malignant cell lines a stimulating cell proliferation was noticed resulting from movement of OTR out of caveolae onto lipid rafts accompanied by activation of alternative signal transduction pathways (39).

There have been few investigations into the sensitization as well as desensitization of OTRs (40–42) and the activity of mRNA-transcription of the OTR (43). The human OTR seems to be quickly (5–10 min) internalized after activation (17)—with both β-arrestin (44) and clathrin (41) identified as being important in the internalization process. Newly discovered aspects of G-coupled receptors have been gaining increased attention such as the possibility of dimerization activation and biased activation (45, 46). To date, there have been limited studies investigating the possibility of OTRs occurring as homo- or heterodimers (with the AVP-receptor) and oligomers (47, 48) which opens up novel approaches for the development of targeted OT-agonists and -antagonists (49) and should receive more attention in the future. Similar attention should be given to exploring biased activation such as for example atosiban (more in chapter “Oxytocin-agonists and –antagonists”) (50).

Oxytocinases such as cystinyl aminopeptidase (CAP) [also known as insulin-regulated aminopeptidase (IRAP) or human placental leucine aminopeptidase (PLAP)] are enzymes which cleave OT (making it inactive) and thereby attenuate the effect of OT over time (51). One study found that androgen levels seemed to regulate a putative member of the oxytocinase family of proteins in the epithelial cells of the rat prostate (52). One study showed that prostatic OT-levels as well as IRAP-specific-activity increased in the rat prostate after treatment with the alpha-1-blocker doxazosin which is used in the treatment of BPH. This might point to the so far underappreciated role of oxytocinases in regulating OT-levels (53).

## Oxytocin Systemic and Local

OT as well as AVP are known to be synthesized in the hypothalamus as part of a larger pro-hormone (with carrier protein neurophysin I for OT and neurophysin II for AVP), stored in the posterior pituitary gland and released into the blood stream as a hormone. Neurophysin is responsible for proper packaging and storage of OT before systemic release (17, 54).

Additional tissue specific local production of OT has been suggested to occur in multiple organs (55) where local OT-levels surpass the plasma OT-levels (10). Local production of OT in the human male reproductive tract has been identified via immunostaining for neurophysin I, radioimmunoassay or the detection of mRNA of the OT-gene in the testis (56) and prostate (54). However, multiple studies in different animal species have shown controversial OT- as well as OTR-expression (57) (Table 1). For example, a study in the marmoset monkey using immunohistochemistry staining (IHC) and detection of mRNA only found OT and OTR being synthesized in the testis but found no convincing evidence for local synthesis in the epididymis or prostate (61). Studies in rams (76) suggested that there is no local production of OT in the epididymis, but that the OT produced by the testis is resorbed in the epididymis (predominantly in the caput). Available evidence suggests this is a maturity related process, since circulating OT-levels in rams of different age groups did not alter, but testicular OT concentrations significantly increased with onset of spermatogenesis (with the main staining for neurophysin I found in Leydig cells, only weak staining in Sertoli cells) and OT was found to be present throughout the epididymis declining in concentration from caput to cauda in all but pre-pubertal animals. AVP-levels did not change in relation to maturation (69). A ligation experiment of efferent ducts in rats (thereby cutting off an OT supply by the testis) suggested that OT in the caput epididymis might not be exclusively from the testis (75). Interestingly, androgens appear to inhibit OT-synthesis in the rat epididymis (75). In stallions no evidence for local production of OT in either testis or epididymis was found (72). In the marsupial bandicoot immunoreactive oxytocin and mesotocin were present in the ventral prostate but only oxytocin was found in the testis (73).

**Table 1:** Original: Expression and localization of OT and OTR in different species throughout the male reproductive tract.

		**OT**	**OTR**
Testis	Human	**−** OT (cDNA library screening and NB) (58) **Ø** OT (PCR) (58) **+** OT and nI (RIA) (56)	**+** (WB) (59) **+** in Leydig and Sertoli cells (IHC) (59) **+** (RT-PCR) (60)
	Marmoset	**+** OT and nI in Leydig cells (IHC) (61) (**+**) OT and nI in Sertoli cells (IHC) (61) **+** mRNA (RT-PCR) (61)	**+** (RT-PCR) (61) **+** in Leydig cells (IHC) (61)
	Macaque		**+** (WB) (59) **+** in Leydig and Sertoli cells (IHC) (59)
	Mouse		**+** (RT-PCR) (62)
	Rat	**+** OT and nI (RIA) (56) **+** OT, but **−** nI in Leydig cells (IHC) (63) **+** OT in interstitial cells (RIA) (64) **+** OT in Leydig cells (IHC) (65) **+** OT in Leydig cells (RIA) (66) (**+**) OT mRNA (PCR) (67)	**+** (WB) (54)
	Sheep	(**+**) probably in Sertoli cells mRNA (NB) (68) **+** OT and nI in Leydig cells (IHC) (68) **+** OT (RIA) (69) **+** OT and nI (IHC) (69) **+** nI (WB) (69)	**+** (WB) (70) **+** in Leydig and Sertoli cells (IHC) (70)
	Goat	**+** mRNA (RT-PCR) (71) (**+**) OT and nI (IHC) (71) **+** OT and nI in Sertoli cells (IHC) (71)	
	Cow	(**+**) probably in Sertoli cells mRNA (NB) (68) **+** OT and nI in Leydig cells (IHC) (68)	
	Horse	**+** OT, but **−** nI (IHC) (72)	
	Rabbit		**+** (RT-PCR) (60)
	Marsupials	Bandicoot: **+** OT (not mesotocin) in Leydig cells (IHC) (73)	Wallaby: **+** (RT-PCR) (74)
Epididymis	Human		**+** (RT-PCR + WB + IHC) (60)
	Marmoset	**−** OT and nI (IHC) (61) (**+**) OT mRNA (RT-PCR) (61)	**+** mRNA (RT-PCR) (61) (**+**) (IHC) (61)
	Macaque		**+** (WB) (59)
	Rat	**+** OT (declining from caput to cauda) (IHC + RIA) (75)	
	Sheep	**+** OT (declining from initial segment or caput to cauda) (IHC) (69, 76) **Ø** nI (IHC) (69) **+** nI (WB) (69) **−** nI (IHC) (76)	**+** (WB) (70) **+** (IHC) (70)
	Cow		**+** (IHC) (77) **+** (RT-PCR) (77)
	Horse	**+** OT (IHC) (72) **−** nI (IHC) (72)	
	Rabbit		**+** (RT-PCR) (60)
	Marsupials		Wallaby: **+** (RT-PCR) (74)
Vas deferens	Marmoset	**−** OT mRNA (RT-PCR) (61) **−** OT and nI (IHC) (61)	**+** mRNA (RT-PCR) (61) (**+**) (IHC) (61)
	Rabbit		**+** (RT-PCR) (60)
	Sheep		**+** (IHC) (70)
Prostate	Human	**+** nI (IHC) (54) In epithelial + stromal cells: **+** OT (intensity dependent on disease) (IHC) (36, 54) BPH- and cancer tissue: **+** nI (WB) (54)	**+** (WB) (59) **+** (RT-PCR) (60) **+** (IHC) (38) In epithelial and stromal cells: **+** (WB) (39, 54) **+** (IHC) (36, 54) **+** (IF) (36, 39) BPH- and cancer tissue: **+** (IHC) (36, 38) **+** (WB) (54) **+** (IF) (39)
	Marmoset	**−** OT and nI (IHC) (61) **−** OT mRNA (RT-PCR) (61)	**+** mRNA (RT-PCR) (61) (**+**) in epithelial cells (IHC) (61) **−** in stromal cells (IHC) (61)
	Macaque		**+** (WB) (59)
	Rat		**+** (RT-PCR + WB) (78) In epithelial and stromal cells **+** (IHC + ARG) (78)
	Rabbit		**+** (RT-PCR) (60)
	Marsupials	Bandicoot: **+** OT (+ mesotocin) (IHC) (73)	Wallaby: **+** (RT-PCR) (74)
Seminal vesicles	Marmoset	**−** OT and nI (IHC) (61)	**−** (IHC) (61)
	Rabbit		**+** (RT-PCR) (60)

*(+ = positive; **−** = weak positive; **+** = negative; **Ø** = inconclusive; nI, neurophysin I; IHC, immunohistochemistry; IF, immunofluorescence; (RT-)PCR, (real time) polymerase chain reaction; RIA, radioimmunoassay; NB, northern blot (hybridization); WB, western blot; ARG, autoradiography)*.

## The Relevance of Possible Crosstalk Between Oxytocin and Arginine Vasopressin

A multitude of studies have investigated the selectivity of the neurohypophysial hormones and have shown crosstalk between the hormone OT and the AVP-receptors, as well as AVP and the OTR using a variety of techniques (e.g., IHC, immunofluorescence, western blot) in different species (5, 12, 79). Importantly, these results taken together indicate that experiments conducted in for example rodents, might not translate to humans. Existing data suggest that AVP has a similar affinity to OT- and AVP-receptors whereas OT has a higher affinity to its own receptor than to AVP-receptors. AVPR1A might also play an important role in OT-mediated contractility as demonstrated in human myometrium (80) and the “ejaculatory tissue” (prostatic urethra, bladder neck and ejaculatory duct) of rats and rabbits (81). The similarities of the two nonapeptides and their receptors, and their demonstrated potential to crosstalk underpins current interest in finding more selective OT- and AVP-agonists and -antagonists especially when administering the drug systemically (82). Nevertheless, the classic roles of both peptides are still preserved; where OT is associated with contractile effects and social bonding whereas AVP is associated with water homeostasis and blood pressure regulation. OT acting through AVP-receptors in addition to its own receptor, might account for some of the observed OT side effects because of their possible relation to homeostasis (headache and dizziness). This cross-reactivity may also explain why, although AVP was described to show similar contractile potential as oxytocin in the male reproductive tract, it might also lead to more severe side effects because of its more prevalent effect on the kidney and vasculature. These observations provide further weight for the development of highly specific OTR-agonists and -antagonists. Still there is at least one AVP-antagonist that has shown some contractile potential (SR 49059) in the male reproductive tract (81) (more in chapter “Oxytocin-agonists and –antagonists).”

## Receptor Distribution and Consideration of Colocalization

Only one OTR-isoform is known to be expressed throughout different tissues. It has been demonstrated by western blotting (WB), real time polymerase chain reaction (RT-PCR) or IHC that the OTR in the human male reproductive tract is present in the testis (59, 60), epididymis (60) and prostate (38, 59, 60). The OTR was further localized via IHC, immunofluorescence (IF) and WB to the interstitial Leydig cells and Sertoli cells of the testis (59) and the epithelial and stromal cells of the prostate (36, 39, 54). Although another study in the marmoset monkey using IHC and detection of mRNA only found OT and OTR to be synthesized in the testis but found no convincing evidence for being synthesized in the epididymis or prostate (61). In the tammar wallaby mesotocin receptors were found in the prostate, but not in the testes (74). In rams OTRs were found in Leydig as well as Sertoli cells and throughout the epididymis (70). Some hypotheses state that OTRs are present in the epithelium of the epididymal duct and that OT partially mediates its contractile effect in the epididymis by regulating the release of endothelin-1(ET-1) (60, 83–85) which in turn is estrogen-dependent (84, 86). Furthermore, estrogen might influence epididymal contractility by upregulating the shared downstream signaling pathway (RhoA\ROCK) of the OTR and ET-1-receptor (87). The role of estrogens in the oxytocin system has been reviewed (88) and some hypothesize that estrogens modulate the expression of the neuropeptide gene for oxytocin (89, 90). One study found that OT and OTR are present in a subpopulation of GnRH neurons and OT might therefore influence neuronal activity centrally (which was independent of estrogen) (91). Androgen-binding protein and OT were colocalized in the reproductive tract of male rats (92). Androgen receptor and OTR colocalization was upregulated in androgen-independent human prostate cancer cells (39).

In myometrial cells of the uterus OTRs were found to be co-expressed with the β2-adrenergic-receptor and they seem to interact by both signaling via the ERK 1/2-pathway (93, 94). The sensitivity of myometrial cells for OT changes throughout parturition (95) and might also shift coupling from Gα_q/11_ to Gα_i_ (96, 97).

Interestingly, OTRs have also been found to be expressed in the endothelial cells of human vasculature (98) which might explain some of OT's side effects.

## Oxytocin in the Testis

**Figure 5:**
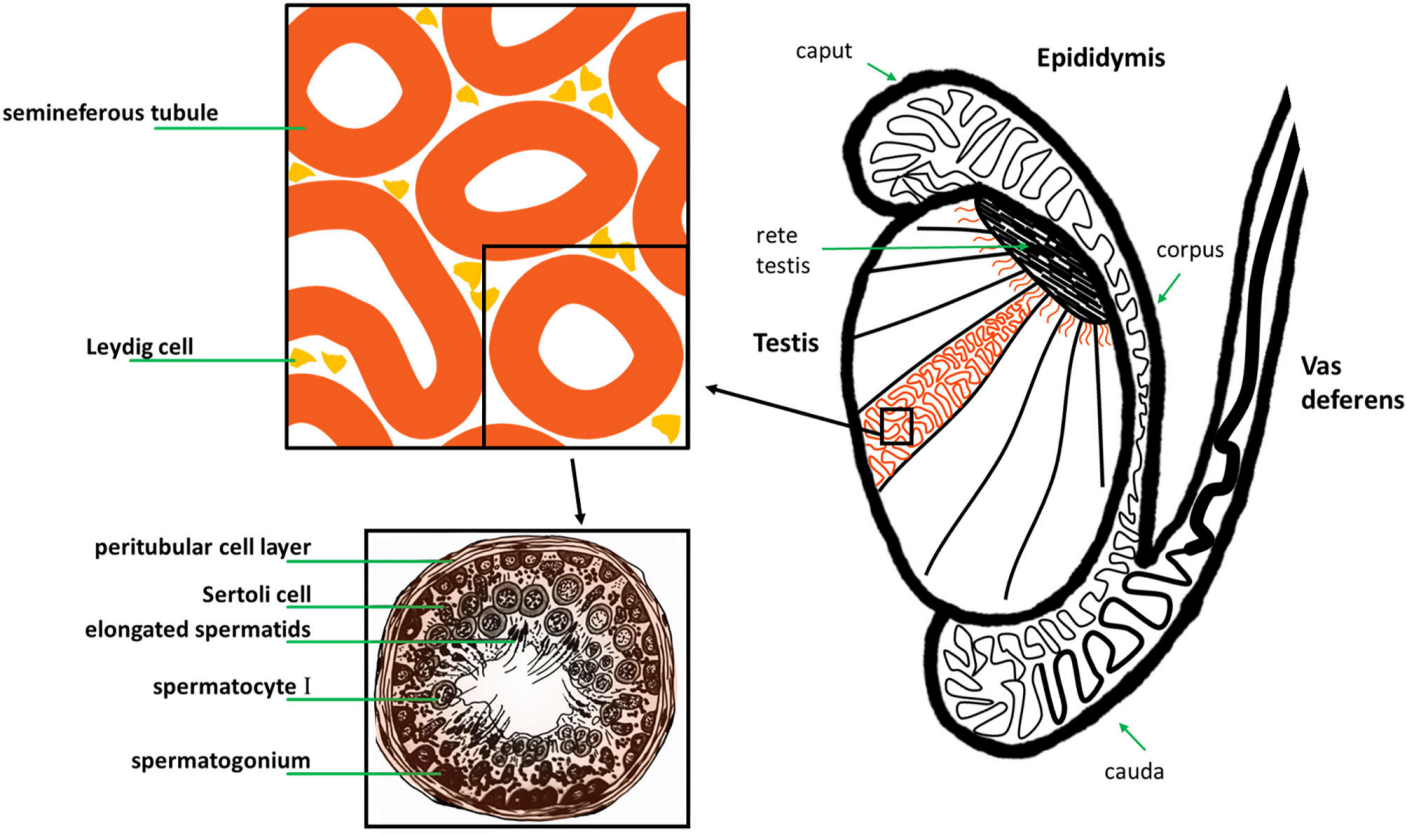
Original: Anatomy of the human testis, epididymis and vas deferens, and histology of a human seminiferous tubule.

All mammals (including the human) possess two testes which are responsible for sperm and androgen production. In the human and most mammals, the testes are externalized in the scrotum. The epithelium of the seminiferous tubules mainly consists of spermatogenic cells and Sertoli cells surrounded by a smooth muscle layer. In between seminiferous tubules lay connective tissue and interstitial cells, Leydig cells (source of androgen production) (Figure 5). Sperm production originates from the germ cells in the epithelium of the seminiferous tubules (spermatogonia). It can be divided into three phases which are preserved throughout the different species: proliferation and differentiation of spermatogonia (Type A and B), meiosis and spermiogenesis. These phases can be histologically distinguished into more or less stages (for example 14 stages in the rat, 6 in the human) (99). In the human there are multiple stages of spermatogenesis in each cross-section of seminiferous tubules whereas in the rat for example there is only one stage per seminiferous tubule section which also influences the contractility of that segment (100). This might have to do with contractions playing a role in the shedding of the “finished” sperm cells into the lumen. There are several factors which contribute to propelling sperm out of the testis and into the epididymis: contractions of the testicular capsule, contractions of the seminiferous tubules and fluid flow. These were also found to differ depending on the species (101) (Table 2).

**Table 2:** Original: Overview of the effect of OT on the male reproductive tract (**+** = increasing; **−** = decreasing; **Ø** = no effect).

	**Effect on smooth muscle contractility**	**Effect on cell proliferation**			**Other effects**
Testis	**+** mouse (102) **+** rat at spermatogenesis stage VII-VIII (100) **Ø** rat at spermatogenesis stage IV-V (100) **Ø** rat, rabbit (81)	Spermatogenesis: **+** rabbit spermatogonia (103) **Ø** mouse (104) **+** mouse (62)			After OT-addition: Less degeneration of spermatocytes during meiosis in rats (103) **+** spermatozoa output in sheep (70, 105) **+** OTR-expression (62)
Epididymis	**+** rat caput (106, 107) **Ø** rat caput (108) **+** cow caput (77) **−** cow corpus (77) **+** rabbit cauda (109) **+** sheep cauda (110) **Ø** rat cauda (108) **+** mouse cauda (111, 112) **−** cow proximal cauda (77) **+** cow mid- and distal cauda (77) **Ø** rat, rabbit (not specified which part) (81)				Tissue that had been blocked by an α-adrenergic-antagonist: **+** ram cauda (while norepinephrine could not) (110)
Vas deferens	**+** sheep (110) **Ø** rat, rabbit (81) **Ø** cow (77) **−** rat (113)				
Prostate	**+** human (BPH tissue) (114), **+** rat, dog, guinea pig (114, 115) **Ø** rat, rabbit (81)		Without gonadal steroids	With gonadal steroids	5α-reductase-activity: **+** PrEC but not in PrSC (116)
		Human prostatic stromal cells (PrSC)	**−** (2) **+** (36)	**−** (2)	Prostate size after OT injection: **+** in castrated rats (117, 118) **+** in mice (36)
		Human prostatic epithelial cells (PrEC) PrSC (co-cultured with PrSC)	**Ø** (2) **+** (36) **Ø** (2)	**Ø** (2) **−** (2)	OTR-expression: **+** in BPH-tissue (36, 38, 59, 119) **+** with age (37, 38) **+** in cancer tissue (120) **−** after OT-addition in PC-3 cells (39) **Ø** after OT addition in PrEC (39)
		Androgen independent malignant cells (PC-3)	**+** (39) **Ø** (2, 120)	**+** (2) **Ø** (39)	OT concentration: **+** in BPH-tissue (36, 54, 121) **+** in cancer tissue (120) **+** in serum of BPH-patients (36)
Seminal vesicles	**Ø** rat (122) **+** rat, dog, guinea pig (115) **Ø** rat, rabbit (81)				

Oxytocin appears to increase contractions of seminiferous tubules in mice (102) which differed depending on the stage of spermatogenesis in rats (100) and therefore could play a role in facilitating the shedding of the haploid spermatozoa into the lumen. One study found that OT failed to elicit tonic contractions in the testes of rats and rabbits (81). OT was found to increase fluid flow and number of spermatozoa in the rete testis of rams (70, 105) and accelerated the arrival of first sperm into the epididymis in pre-pubertal rats (104) while, importantly, an OT-antagonist delayed it (123). AVP has been suggested to have an effect on seminiferous tubule contractility by acting via the AVP1A-receptor whereas OT might be acting through an unidentified subtype and not the OTR or AVP1A-receptor in the rat (124). Some studies postulated that OT has no direct effect on spermatogenesis since OT-knockout mice or testicular OT-overexpressing mice had no detectible differences in testicular morphology or germ cell development compared to wild type controls (104). The most recent study however, found that OT increased the number of spermatocytes and round spermatids, which was attributed to the increased proliferation of germ cells in the testis of pre-pubertal mice (62).

There are two cell types identified as the source for OT-synthesis in the testis: Leydig cells and Sertoli cells (Table 1). Luteinizing hormone (LH) was found to stimulate OT production in Leydig cells (66) independently of testosterone (125). OT was found to stimulate testosterone production in mice (62), goats (71), and in pre-pubertal rats (126). In cell culture experiments of rat Leydig cells OT was either found to stimulate testosterone production (127) or have no effect (128). AVP appeared to stimulate testosterone production in rat Leydig cells for up to 5 h, but not thereafter, while in the presence of human chorionic gonadotropin (hCG) AVP inhibited testosterone production beyond 24 h of culture (128). Arginine-vasotocin (another neurohypophysial hormone homologous to OT and AVP) was capable of directly inhibiting Leydig cell androgen biosynthesis (129). Interestingly, OT had no effect on LH-induced testosterone production in the rat Leydig cells and AVP decreased LH-induced testosterone production (127). Long-term application of OT through an intratesticular implant in rats lead to a reduction of testicular and plasma testosterone but elevated DHT-levels. The number of Leydig cells, plasma LH- or OT-concentrations as well as epididymal sperm count did not change (130).

Because OT is secreted in the testis, has an effect in the testis itself and is regulated by other factors regulating gonadal function, it is suggested to consider OT as a male gonadal hormone (131).

## Oxytocin Related to Emission and Copulation

Running down alongside the two testes are the two epididymis. The infertile sperm cells gain fertility and motility while traveling through the long coiled epididymal duct (human 6 meters, rat 1 meter) which is tightly packed and can be classified into initial segment, caput, corpus and cauda (Figure 5). This can be further classified into a number of segments [human: 10 (132), mouse: 10, rat: 19 (133)] with each exhibiting different gene expressions and micromilieus. The now fertile and motile sperm is stored in the last segment of the cauda epididymis until release during the emission phase of the ejaculatory process. It then travels through the adjacent vas deferens up through the abdominal cavity and around the bladder emptying into the upper part of the urethra. The sympathetic neurons play the predominant role in the male ejaculatory process. Their nerve terminals secrete primarily norepinephrine (134–136). As far back as the 60 and 70s OT has been investigated for its effect on ejaculate volume, sperm count and higher plasma OT-levels, found after stimulation of reproductive organs.

Debackere et al. were one of the firsts to propose that plasma OT rises after manual stimulation of male and female genital tracts in sheep (137) and also noticed that injection alone with a synthetic OT did not lead to an emission in the male. After that multiple studies showed a rise of OT levels after manual stimulation or sexual arousal/orgasm in male and female ponies (138), bucks (139), bulls (140) and men and women (141–146). It was also noted that baseline OT-levels were either higher in women then in men (144, 147) with higher blood flow to the pituitary gland during orgasm in women (148) or the same in men, women and pregnant women (149, 150). In mares OT-levels seemed to fluctuate depending on the menstrual cycle (highest around ovulation) (138). In an earlier study OT-levels in women were reported not to increase during pregnancy or labor but only spiked after seeing the baby (149), whereas more recently it was found that plasma OT-levels rose progressively over the course of pregnancy, with high pulses of OT being released during labor (151). It was also found that OT- but not AVP-receptors increase during pregnancy on myometrial membranes in humans (152–154). OT-binding sites and the regulation of OT-binding was found to fluctuate depending on oestrous cycle and during gestation in the rat (155). One of the reasons for contradictory data on OT- and AVP-plasma levels might be due to difficulties involved in using bioassays (e.g., different tissue sensitivity, low circulating levels) (156).

AVP-levels appeared to be higher in men (147, 157) and were either reported to stay at the same level throughout sexual intercourse (141) or as going up only shortly before ejaculation (145) in men. Prolactin (141) and epinephrine (158) were also noted to increase around orgasm and in relation to OT.

There are big differences in ejaculates depending on the species (ejaculate volume, proportion of spermatozoa to seminal fluid, proteins comprising the seminal fluid, total sperm count, morphology of the sperm cells, etc.). Interestingly, the semen quality (e.g., motility, sperm count, number of deformations) that would classify “normal” in humans would be classified as too low in animals. This might play a role when testing male contraceptives since they might prove much more effective in the human than an initial study in animals may suggest.

Studies were performed which specifically investigated the effect of OT on ejaculate volume, sperm count, and other parameters involved in ejaculation. In studies conducted with multiple mounts and ejaculates collected per experiment it was found that intravenous injection of OT in rabbits (159, 160) increased sperm count and sperm volume in the first ejaculates, which then decreased in subsequent ejaculates resulting in a total sperm count no greater than the one in the control. This would suggest that after intravenous administration of OT the storage of sperm (in cauda epididymis) is simply emptied out quicker than in control. In rams similar results (161) suggest an influence of OT only on the emptying of the stored sperm in the epididymis but also found an increase of abnormal spermatozoa in ejaculates after week 6 of the experiment. Since normal spermatogenesis takes around 6 weeks in the ram this would suggest that the contractile properties of OT might either lead to a premature shedding of spermatozoa in the testis or reduced time spent traveling through the epididymis to gain full maturity of the spermatozoa. One study looking into OT's effect on sperm count in rabbits suggested that intravenous injections might decrease the number of spermatocytes undergoing degeneration during meiosis in the testes or increase mitotic activity of the spermatogonia (103). A decreased number of Sertoli cells was also noted. The author hypothesized that OT may cause a hypersecretion of gonadotrophic hormones resulting in an inhibitory effect on spermatogenesis. In bulls one study found that OT enhanced sperm output in first ejaculates of electro-ejaculated bulls without altering daily sperm production or seminal quality (162), while another found that OT significantly increased the percentage of motile spermatozoa and sperm velocity compared with saline controls (163). In the rat it was found that OT over a short period of time with only a few ejaculates improved sperm count (164), whereas a more recent study (165) found that OT dose-dependently reduced sperm number in vaginal ejaculates, suggesting post-testicular oligospermia. There was no effect on fertility, although the authors found significant amounts of sperm in bladders of OT-injected animals.

Additionally, an experiment with an OT-antagonist showed a decrease of sperm count and semen volume over time in sheep (166) and another OT-antagonist inhibited male copulatory behavior (167). Interestingly, a study in rams showed only an increase of sperm count and semen volume in response to OT but not after norepinephrine administration (168).

Again AVP was also evaluated for its effect and found to have either none (in sheep) (166) or an increasing effect on sperm count and semen volume (in rabbits) (169).

In humans 4 independent studies found no effect of OT on sperm count, semen volume, motility or time until ejaculation in healthy or oligospermic men (170–173), however one case of male anorgasmia was successfully treated by intracoital application with intranasal OT (174). One reason for OT to be found less effective in human clinical trials might be the psychological component of being aware of the experiment, especially with respect to the link between intimacy and human sexual function. Interestingly, Goverde et al. found OT to be present in the semen of vasectomized men suggesting that the OT in human semen does not only originate from the testis or epididymis (172).

Due to its role in propelling the sperm forward during the emission phase of the ejaculatory process, the epididymis was studied for its contractile responses to OT and AVP (Table 2). Special interest was paid to the contractile effect of the cauda epididymis which was found to either increase in frequency and amplitude in the mouse (111, 112), rat (107), rabbit (109), and ram (110) or to have no effect in the rat and rabbit (81). One study in the rat found that OT had little to no effect, while AVP increased both frequency and tension in caput and cauda epididymis (108). Other studies found that both OT and AVP had an increasing effect on the contractility of the initial segment of the rat epididymis (106) and AVP to be less effective than OT in ram cauda epididymis (110). Interestingly, Knight also found that in ram cauda epididymis OT was able to contract tissue that had been blocked by an α-adrenergic-antagonist while norepinephrine could not (110). A study in bulls demonstrated different reactions to norepinephrine and OT depending on the area of the epididymal duct investigated (77). Both had similar positive effects in caput and cauda, whereas OT had a relaxing effect on corpus and proximal cauda epididymis. Norepinephrine had a much bigger effect on the vas deferens of the bull than OT (77). Other studies found OT to have either no effect in the vas deferens of rat and rabbits (81), depress the contractile response in the rat (113) or increase contractility in the ram (110).

OTRs were found in both human and rabbit corpus cavernosum (175, 176) and OT-levels in cavernous blood increased differently to circulating OT-levels depending on penile erectile states (177). Evidence also suggests that OT plays a role in inducing erections (178–181), decreasing number of intromissions before ejaculation and modulating post-ejaculatory and/or sexual behavior (139, 182, 183) by affecting the central nervous system directly. Despite the clear evidence for a central role of OT in the mechanics of sexual function, the main focus of a multitude of studies investigating the effect of OT on the central nervous system itself has been associated with e.g., anxiety, autism, pair bonding. Still one case of male anorgasmia was successfully treated by intracoital application with intranasal OT (174), while OT-antagonists have shown to inhibit ejaculation both peripherally and centrally in rats (184, 185).

Some studies found that OT in the seminal fluid might even help direct sperm transport by inducing uterus contractions leading to the ovary carrying the dominant follicle (186, 187).

Despite all of OT's involvement in male and female reproductive functions OT-deficient mice had no defects concerning mating behavior, conception, pregnancies, litter size and labor (188), suggesting that either OT is only supporting reproductive functions or maybe that another OT-like agent is acting at the OTR in OT-deficient mice. However, OT was found to be essential for milk ejection (189, 190). Without OT milk will still be secreted but will no longer be ejected into the collecting ducts of the mammary glands which results in a lack of milk available for breastfeeding (188) and ultimately the death of the offspring.

## Oxytocin in the Prostate

In addition to the sperm cells the ejaculate also consists of seminal fluid which is produced by one or multiple accessory sex glands depending on the species. The human possesses the prostate, seminal vesicles and the bulbourethral glands with the prostate contributing the most fluid. The rat possesses the seminal vesicles, prostate, coagulating glands, bulbourethral glands, and preputial glands, whereas the cat only has one accessory sex gland, the prostate (191). Since the prostate is the most important accessory sex gland in the human and is prone to diseases such as benign prostatic hyperplasia (BPH), prostatitis and cancer (being the most often diagnosed human cancer overall) it is a very important part of the male reproductive tract. The prostate however is very different in morphology, histology and physiology throughout different species. Otters for example do not possess a prostate at all and the prostate of marsupials appears to undergo seasonal changes correlated to seasonal breeding. The human prostate is very close to the bladder neck, tightly surrounds the urethra and possesses a dense fibromuscular outer layer.

When investigating the effect of oxytocin on the prostate, two differentiations are most often made: the effect on contractility and the effect on proliferation (with or without interfering with androgen levels) (Table 2). Localization of the OTRs in either epithelial cells or stromal cells or both apparently varies depending on the species (Table 1). In organ bath studies OT has been shown to have an increasing effect on the spontaneous prostatic contractions as well as prostatic tone in guinea pig, rat, dog and human prostate (114). The OT-induced contractions in the prostate were characterized as being slower and longer lasting than those induced by norepinephrine (115) in the guinea pig, rat and dog. While the first study found AVP to be less effective than OT, the second study found it to be more effective. More recently one study found no effect of either OT nor AVP on the tone or contractility of the prostates of rats and rabbits (81).

**Figure 6:**
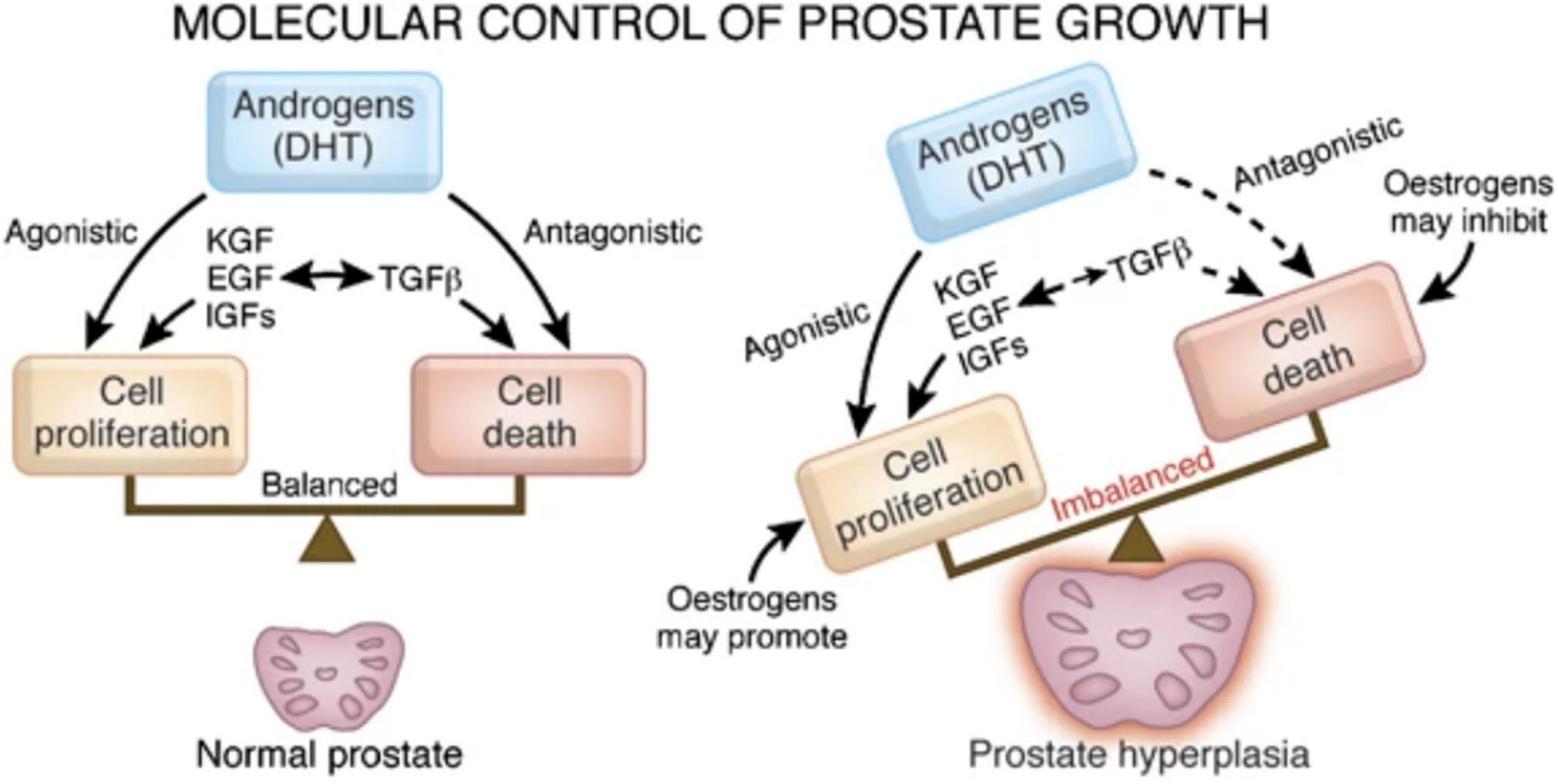
Re-published: Steroids involved in keeping the balance of human prostatic growth. Interaction of OT and androgens as well as a direct effect of OT is not displayed by Roehrborn (194). Reprinted by permission from Springer Nature: Nature, International Journal of Impotence Research, Pathology of benign prostatic hyperplasia, Roehrborn (194) https://www.nature.com/articles/ijir200855.

In addition to the human only a few other species (dog, chimpanzee) develop the multifactorial disease BPH. In the human this disease is often associated with lower urinary tract symptoms. There are artificially induced rat BPH-models to create a hyperplastic-like prostate model. BPH in dogs and rats however fails to elicit the lower urinary tract symptoms often associated with BPH in the human. One reason for that might be because they lack the dense fibromuscular outer layer (that the human possesses) and therefore the overgrowth of the prostate can proliferate outward, not compressing the urethra and restricting urine flow. The specific cause of the multifactorial disease BPH is still unclear but androgens (as well as estrogens) have long been suspected to play an important role in BPH genesis and development (192), although there have also been contradicting studies (193). In the prostate ~90% of androgens are in the form of dihydrotestosterone (DHT). The enzyme 5α-reductase converts testosterone to DHT. Androgens and estrogens are involved in regulating a multitude of growth factors in the prostate to keep a balance of proliferation and cell death (Figure 6) (194).

In case of BPH prostatic DHT-levels (as well as testosterone-levels) are observed to be higher than in normal or cancerous tissue (195), therefore one treatment option for BPH aims to reduce DHT levels by decreasing 5α-reductase-activity with 5α-reductase-inhibitors, such as finasteride.

In the human prostate OT increased 5α-reductase-activity was only found in the epithelial cells but not in the stromal cells (116). In observing the effect of subcutaneous injections of OT on the ventral prostate of rats over a period of 10 days, only in the castrated not the intact rats was OT found to have a BPH-like influence (enlarged prostatic volume, folded epithelium, etc.) (118). Later it was found that the OT-effect in the castrated rats was due to stimulating mitotic activity and diminishing apoptosis of the secretory cells in the ventral prostate (117). More recently a study also showed an enlargement of prostates in intact mice after 2 weeks of intraperitoneal OT-administration (36).

In the rat prostate OT-treatment temporarily increased 5α-reductase-activity (196). After 3 days of OT-administration 5α-reductase-activity and prostatic DHT-levels are both raised, but after the following 4 days both values returned to normal (78, 118). An increase of androgens in the rat might downregulate the level of prostatic OT (and numbers of OTRs), while a decrease of androgens might upregulate prostatic (not systemic) OT which implicates OT in a negative feedback role in the regulation of androgens in the rat prostate (121, 197).

In consideration of BPH in dogs and men it has been found that prostatic OT-concentrations are significantly higher in BPH-tissue (121). Using cultured human BPH-tissue it was also found that testosterone, DHT and a synthetic estrogen [diethylstilbestrol (DES)] all increased the secretion of OT after 3 days (198). Also increased serum and prostatic OT-levels were detected in cases of BPH (36) and\or prostatic cancer (120). Therefore, OT was suggested as a marker for proliferative alterations of the human prostate. Interestingly, less OT-staining could be observed in the malignant tissue compared to BPH in humans (54). Not mentioned in Figure 6, in human cell culture experiments OT directly inhibited prostatic stromal cell proliferation but had no effect on prostatic epithelial cells (2). OT itself had no effect on malignant cells, however in combination with testosterone it stimulated cell growth (2). In contrast, more recently OT or androgens alone were found to have a proliferative effect on androgen-independent cancer cells but together they had no effect (39).

Androgen receptor and p21-staining was found throughout the human prostate but did not change with age or BPH (37). OTR-expression in the prostate has been found to be increased in tissue with BPH (59, 119). Androgen receptor and OTR-colocalization was upregulated in androgen-independent human prostate cancer cells (39). An OTR-mediated process coupled to G_i_ was described to change migration of prostate cancer cells (199). The role of OT as an effector in different cancer types including prostate cancer is also being investigated (1).

The species-dependent difference of OT's ability to regulate androgen levels might be related to the fact that rats do not develop BPH spontaneously. There are only very few species that develop BPH (e.g., human, dog, chimpanzee). Perhaps the regulatory feedback mechanism downregulating DHT in rats after a couple of days, which has not been found to exist in the human and the dog, plays an essential role in preventing the development of BPH (196). Interestingly, seasonal changes of prostate size and testosterone secretion seem to occur in the brushtail possum (200). More recently it has been suggested that there might be an androgen-independent correlation of mesotocin (OT-like peptide) and prostatic growth (201).

OT has been proposed to be a paracrine regulator of the prostate since it has been shown to be locally produced in the prostate as well as having a contractile and proliferative effect while being regulated by other prostatic effectors (121).

## Oxytocin-Agonists and -Antagonists

**Figure 7:**
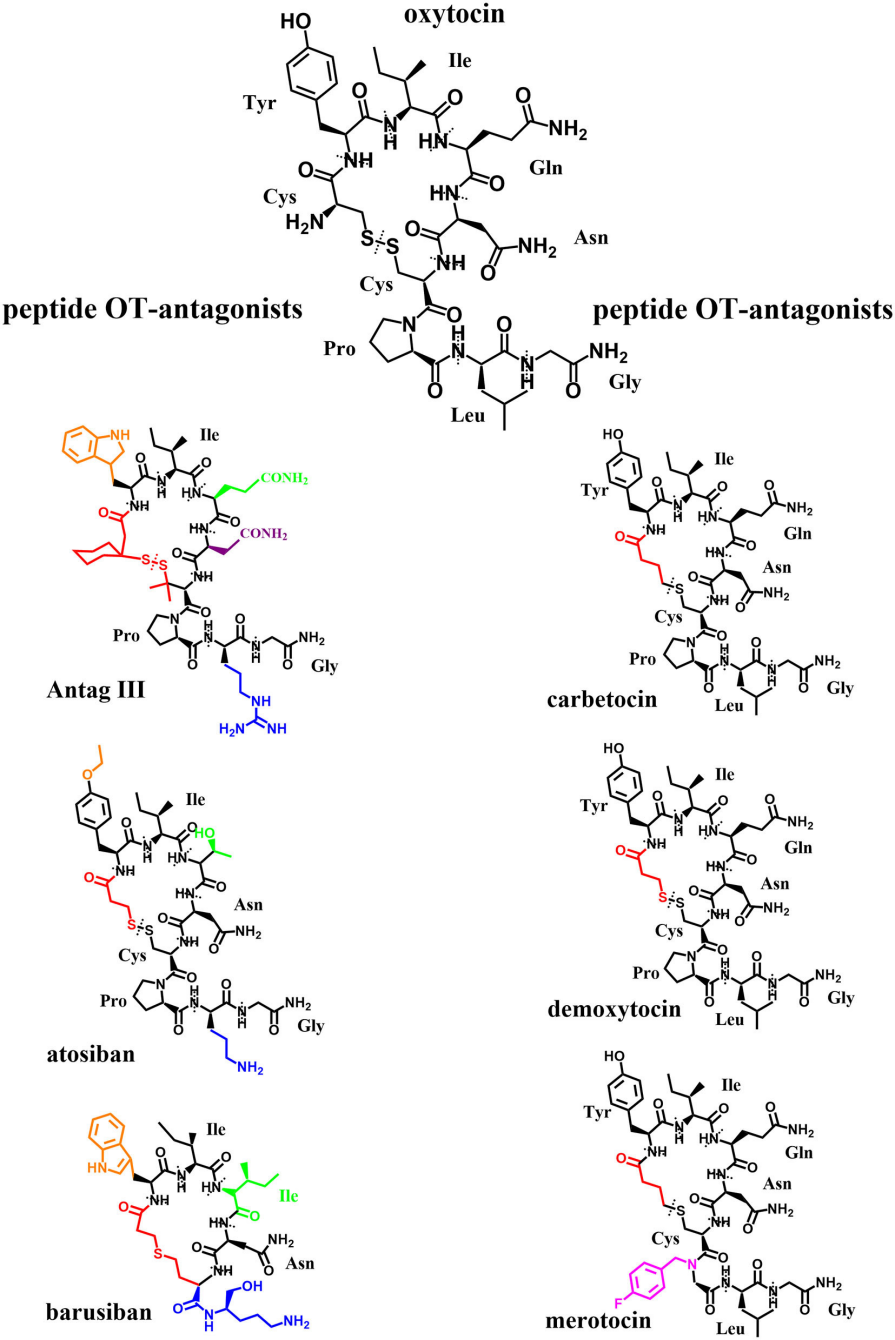
Original: Chemical structures of OT and six peptide OT-agonists and -antagonists. Alterations in respect to OT have been highlighted in different colors. The different amino acids are labeled and separated by dotted lines.

**Figure 8:**
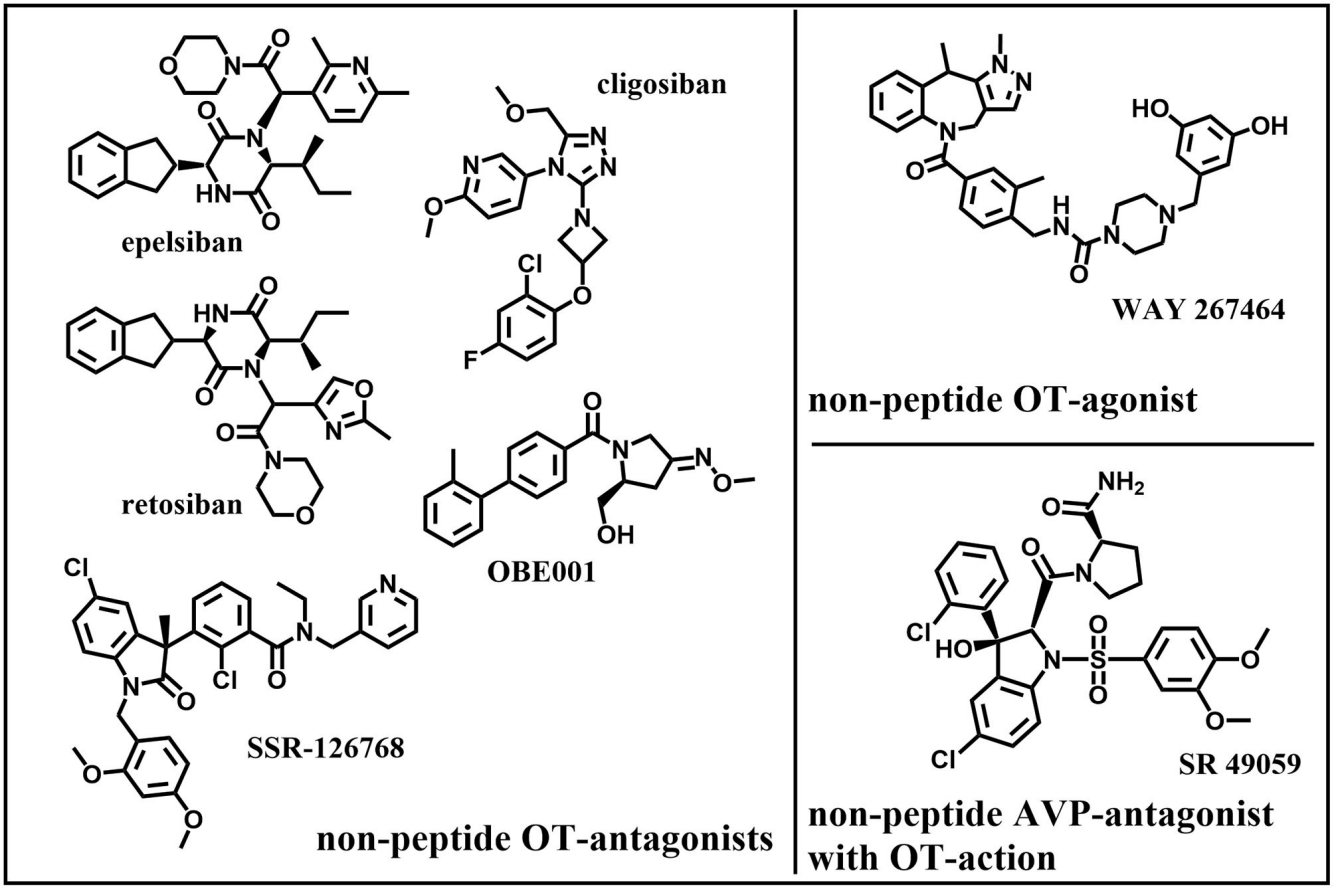
Original: Chemical structures of five non-peptide OT-antagonists, one –agonist, and one non-peptide AVP-antagonist with OT-action.

Due to their potential for pharmaceutical use, OT-agonists and -antagonists have a long-standing history (over 50 years) of being synthesized for use in parturition. However, surprisingly, so few oxytocin-agonists and -antagonists have reached clinical testing and even fewer have been approved for clinical application: success stories include peptide-based and non-peptide small molecules. Coupled with the increased understanding of both the OT- and AVP-signaling pathways, there has been a corresponding increase in interest in the development of new selective OT-agonists and -antagonists as potential treatment options for other conditions outside parturition: diseases of the central nervous system (4, 202), metabolism (3), cardio-vasculature (203, 204), gastrointestinal tract (205), kidney (206), liver (207), bone (208) and other targets within the reproductive tract (10, 209). The differing effectiveness of OT in these diverse tissue targets invites to speculate: It might be due to the tissue-specific occurrence of the OTR as dimer or oligomer, changed signaling pathways due to coupling to differing G-proteins and/or due to colocalization with other receptors. For the successful application of OT-agonists or -antagonists as treatment options, it is important to fully consider different routes of administration to specifically target these organ systems. The development of peptide-based oxytocin-agonists and -antagonists which withstand degradation in the gastrointestinal tract has been challenging (210–212). They also suffer short pharmacokinetic half-lives necessitating continuous application by either intramuscular or intravenous application which is only reasonable for short periods of time. These routes of systemic delivery often lead to undesirable off-target side-effects (e.g., cardio-vasculature), with the exception of the central nervous system due to the blood-brain barrier (BBB) (213). Intranasal applications and antagonists with BBB-penetrating properties have been developed to affect the brain and central nervous system specifically. Therefore, OT-agonists and -antagonists with more desirable physio-chemical and pharmacological properties such as heat-stability (214–216), potency (217), bioavailability (211), long-acting etc. (218), are continually sought-after. While the search for new OT-agonists and -antagonists initially focused on the subtle manipulation of the amino acid sequence and site-specific chemical modification of oxytocin itself (Figure 7) based on rational linkage of bioactivity to structural conformation (219), this has quickly expanded to include structurally dissimilar small molecules (Figure 8) through drug discovery paradigms.

The development and the synthesis of new oxytocin agonists and antagonists has been widely reviewed (220–223). Here we would like to summarize the more important OT-agonists and -antagonists especially with the focus on reproductive health:

Promising agonists:

- The peptide carbetocin is used in several countries to restore uterine tone and prevent hemorrhage after cesarean section or to treat postpartum hemorrhage. Since recent studies show carbetocin not to be dependent on cold storage such as OT, carbetocin might prove essential in postpartum care in lower income countries while showing similar efficacy and safety as OT (224, 225) or even more effectiveness (226–228) with less side effects in clinical trials (229). Carbetocin and its metabolites carbetocin metabolite I and II are shown to display the same binding affinity to the OTR as OT, however carbetocin's maximal contractile effect was 50% lower than OT's, carbetocin metabolite I and II had no contractile effect at all. Interestingly, all three compounds showed antagonistic properties at the OTR *in vitro*, making carbetocin a partial agonist/antagonist and also demonstrated some affinity to bind to the AVPR1A (230).
- The peptide demoxytocin showed no superiority to PGE2-treated women in a clinical trial (whereas OT did) and induced more gastrointestinal side effects (231)
- WAY-267464 is a potent, selective, non-peptide OTR-agonist that showed a similar anxiolytic-like profile as OT (232).

Promising antagonists:

- The peptide atosiban is a competitive AVP/OTR-antagonist by inhibiting OT-induced IP3-release. Atosiban is the only OT-antagonist that is prescribed and is indicated as a therapeutic to delay preterm birth. Recent studies suggest that atosiban shows similar effectiveness in delaying preterm labor compared to β2-tocolytics although it appears that atosiban has less side effects (nausea, headache, dizziness, tachycardia, hyperglycaemia) than β2-tocolytics (233, 234). Studies could not find a significant difference to placebo-treated controls (235). Atosiban was found to have no effect on human sperm motility *in vitro* (236). It has been suggested that atosiban's anti-proliferative effect in some cancer cell lines (including prostate cancer) might be due to a biased agonistic effect where atosiban blocks OT binding to Gα_q/11_ coupling and thereby promotes OT-coupling to Gα_i_ which leads to inhibition of cell growth (50).
- The peptide barusiban is a selective OT-antagonist with a high selectivity for the OTR. Despite being reported to inhibit OT-related contractility as potent as atosiban (237) or even more potent (238, 239), barusiban has failed to show effectiveness in human clinical trials so far (240).
- Retosiban (GSK221149A) is a highly selective, orally active, non-peptide OTR-antagonist that inhibits OT-induced uterine contractions (241) and showed efficacy in human clinical trials (242).
- OBE001 is an orally active, non-peptide OT-antagonist that is tested for management of preterm labor and showed no adverse effects on early embryonic development in the rat model (243).
- The peptide TT-235 (Antag III) is a long-acting, competitive OT-antagonist that may inhibit the uterine response to OT by decreasing OTR-numbers and -affinity and therefore shows a prolonged activity in comparison to OT (244).
- SSR-126768A is an orally active, selective, non-peptide OT-antagonist with a long duration of action as a tocolytic in the management of preterm labor (245).
- Relcovaptan (SR 49059) is an orally active, non-peptide AVP1A-receptor selective antagonist that also showed tocolytic properties in treatment of preterm labor (246) and was able to potently antagonize OT's effect in the rat and rabbit ejaculatory tissues (prostatic urethra, bladder neck and ejaculatory duct) (81).
- Cligosiban is a potent, brain-penetrating, highly selective, non-peptide OT-antagonist that inhibited apomorphine-induced ejaculation in the rat (184). In a human clinical trial however it failed to prove efficacy (247).
- Epelsiban (GSK557296) is a non-peptide OT-antagonist that dose-dependently inhibited ejaculations in rats both peripherally and centrally (185). In a human clinical trial however it failed to prove efficacy (248).

Both (cligosiban and epelsiban) might still prove valuable as a new treatment option in case of premature ejaculation.

## Discussion

OT presents as an effector throughout the male reproductive system. The initial research into OT's contractile effect in relation to reproduction has been shifted to research mainly into OT's proliferative effect. One contributing factor for this shift might be that OT's contractile effect in the human appears to be weaker than in the animal models. Consideration should also be given to a potential psychological effect of oxytocin and/or the psychological influence of being aware of the experiment, especially with respect to the link between intimacy and human sexual function.

Most of the literature on OT- and OTR-expression dates back 20 years or more, and data on OT- and AVP-levels measured in plasma seem unreliable. Especially with the new wave of interest in the OT-system, it seems reasonable to validate these old findings with new investigations taking advantage of advanced techniques (e.g., 3D-imaging, qPCR).

Activation, sensitization and desensitization of the OTR should be investigated further as well as examining oxytocinases as potential therapeutic tools. The suggested occurrence of G-protein coupled receptors as dimers/oligomers and the targeted activation of specific G-protein subunits appear very promising.

Integrating future findings on these topics with old and new knowledge on drug development could help finding highly specific OT-agonists and -antagonists not only for the different tissues in the male reproductive system but for a multitude of organ systems.

Based on all this we feel that OT-agonists could support spermatogenesis and different stages of sperm transport (in the testis, epididymis, uterus). They might also help in managing ejaculatory disorders as a result of treatment with alpha1-blockers for BPH. Brain-penetrating OT-agonists showed potential for treating anorgasmia. OT plasma levels might be a marker for prostate cancer and BPH. OT-antagonists could be useful treatment options for cases of BPH and premature ejaculation by relaxing smooth muscle cells.

## Author Contributions

BS, MW, BE, and RM wrote the manuscript. All authors contributed to the article and approved the submitted version.

## Conflict of Interest

The authors declare that the research was conducted in the absence of any commercial or financial relationships that could be construed as a potential conflict of interest.
